# Towards the development of a wellbeing model for aboriginal and Torres Strait islander peoples living with chronic disease

**DOI:** 10.1186/s12913-017-2584-6

**Published:** 2017-09-15

**Authors:** Carol Davy, Elaine Kite, Leda Sivak, Alex Brown, Timena Ahmat, Gary Brahim, Anna Dowling, Shaun Jacobson, Tania Kelly, Kaylene Kemp, Fiona Mitchell, Tina Newman, Margaret O’Brien, Jason Pitt, Kesha Roesch, Christine Saddler, Maida Stewart, Tiana Thomas

**Affiliations:** 1 Wardliparingga Aboriginal Research Unit, South Australian Health and Medical Research Institute, North Terrace, Adelaide, South Australia 5000 Australia; 2 Wuchopperen Health Service, 22C Evans St, Atherton, QLD 4883 Australia; 3 Wirraka Maya Health Service Aboriginal Corporation, 17 Hamilton Road, South Hedland, WA 6722 Australia; 4 Nunkuwarrin Yunti Inc, 182-190 Wakefield Street, Adelaide, South Australia 5000 Australia; 5 Maari Ma Health Aboriginal Corporation, 428 Argent St, Broken Hill, NSW 2880 Australia; 6 Tharawal Aboriginal Corporation, 187 Riverside Dr, Airds, NSW 2560 Australia; 7 Danila Dilba Health Service, 1/26 Knuckey St, Darwin, Northern Territory 0800 Australia; 8 Winnunga Nimmityjah Aboriginal Health Service, 63 Boolimba Cres, Narrabundah, ACT 2604 Australia

**Keywords:** Aboriginal and Torres Strait islander people, Indigenous, Primary health care, Wellbeing, Resilience

## Abstract

**Background:**

Re-defining the way in which care is delivered, to reflect Aboriginal and Torres Strait Islander peoples’ needs and values, is essential for improving the accessibility of primary healthcare. This study focused on developing a Framework to support the quality of care and quality of life of, as well as treatment for, Aboriginal and Torres Strait Islander peoples living with chronic disease.

**Methods:**

A team of researchers, including thirteen experienced Aboriginal healthcare professionals, came together to undertake this important work. Using a Participatory Action Approach, this study actively engaged people with local knowledge to ensure that the Framework was developed by and for Aboriginal people.

**Results:**

The final Wellbeing Framework consists of two core values and four elements, each supported by four principles. Importantly, the Framework also includes practical examples of how the principles could be applied. National Reference Group members, including community representatives, policy makers and healthcare providers, reviewed and approved the final Framework.

**Conclusion:**

The outcome of this collaborative effort is a Framework to guide primary healthcare services to develop locally relevant, flexible approaches to care which can respond to communities’ and individuals’ varied understandings of wellbeing.

## Background

Although Australia is a developed country with a relatively well funded healthcare system, Aboriginal and Torres Strait Islander peoples experience a similar prevalence of chronic disease to people in developing countries [1]. The burden this places on Aboriginal and Torres Strait Islander communities is well documented, with cardiovascular disease acknowledged as the single leading cause of death, type 2 diabetes currently at epidemic proportions, and rates of chronic kidney disease disproportionately higher in Aboriginal and Torres Strait Islander peoples compared to non-Indigenous Australians [2]. Collectively, these conditions substantially contribute to the 10 to 14 year life expectancy gap between Indigenous and non-Indigenous Australians [3].

Access to appropriate, affordable and acceptable comprehensive primary healthcare is vital for preventing and managing chronic disease [4, 5]. Nonetheless, use of primary care services by Aboriginal and Torres Strait Islander peoples is lower than could be expected given the high burden of disease they face [6, 7]. The obstacles faced by Aboriginal and Torres Strait Islander peoples attempting to access primary healthcare services are many and varied. Appropriate infrastructure, sufficient funding and knowledgeable healthcare professionals are crucial, but these elements alone will not lead to accessible primary healthcare services.

Instead, a holistic approach to health, which is more consistent with many Aboriginal and Torres Strait Islander peoples’ needs and values is needed [8]. Incorporating not only physical and psychological dimensions, health for many Aboriginal and Torres Strait Islander peoples also requires links to culture, Country, community and family [9, 10]. An acknowledgement of the extent to which Aboriginal and Torres Strait Islander peoples continue to be affected by colonisation brought about by formal policies of segregation and exclusion, as well as forced removal from Country and family [11], is also important.

The Kanyini Vascular Collaboration (KVC), a partnership of Aboriginal, Torres Strait Islander and non-Indigenous clinicians and researchers, has been exploring the systemic barriers to primary healthcare for Aboriginal and Torres Strait Islander peoples. One of the more recent KVC studies [12–14] identified numerous opportunities for improving Aboriginal and Torres Strait Islander healthcare services including creating ‘welcoming healthcare spaces’ where community members could feel they belonged, were accepted and understood. Focusing on supporting people to live the life they want despite managing chronic disease and improving the cultural safety of healthcare services were also important. Cultural safety in this sense moved beyond cultural sensitivity (which implies an awareness of particular cultural differences) and cultural competency (which requires an understanding of specific cultural beliefs) to empower the Aboriginal client to define what a culturally safe services means for them [15]. Building relationships which lead to sustained engagement whereby the patients and providers together are able to determine the ways in which care is provided appeared to be at the heart of the type of healthcare that Aboriginal and Torres Strait Islander peoples with chronic disease seek.

In order to consolidate these primary care developments, the KVC undertook a study focused on identifying ways in which primary healthcare services could better support the quality of care and quality of life for Aboriginal and Torres Strait Islander peoples living with chronic disease. This paper outlines the process and outcomes of this facilitated, multi-jurisdictional dialogue with Aboriginal people and their healthcare services which outlined a framework that can be utilised within primary care services to support the wellbeing of Aboriginal and Torres Strait Islander people living with chronic diseases. Additional information on the methods and results can be found on the KVC website [16].

## Methods

**Fig. 1:**
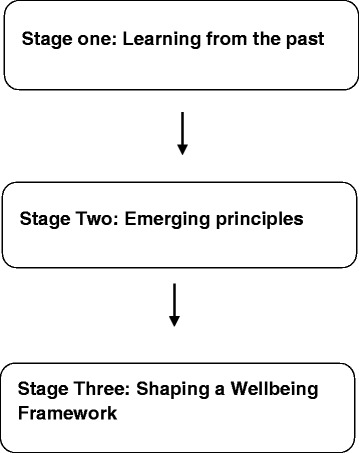
Study Overview

The study consisted of three integrated stages (Fig. 1) undertaken between June 2013 and December 2014. Initially our team consisted of three Aboriginal and three non-Indigenous clinicians and researchers. By the end of the study an additional 13 Aboriginal and Torres Strait Islander Research Fellows (Research Fellows), all of whom were working in Aboriginal Health Services, had joined the team.

Our study was approved by nine ethics committees which represented the range of jurisdiction involved – Aboriginal Health Research Ethics Committee (04–13-533), Western Australian Aboriginal Health Ethics Committee (542), Aboriginal Health & Medical Research Council of New South Wales (980/13), Australian Institute of Aboriginal and Torres Strait Islander Studies (reference number not provided), Central Australian Human Research Ethics Committee (HREC-13-190), Human Research Ethics Committee Cairns and Hinterland, Cape York, Torres Strait and Northern Peninsula Hospital and Health Services (HREC/13/QCH/121–873), Human Research Ethics Committee of the Northern Territory Department of Health and Menzies School of Health Research (2013–2128), Metro South Human Research Ethics Committee (HREC/13/QPAH/526), and Ethics Review Committee Royal Prince Alfred Hospital (X13–0370 & HREC/13/RPAH/518).

A National Reference Group, which consisted of Aboriginal and Torres Strait Islander community members including a number of community Elders, as well as representatives from Commonwealth and State governments, non-government organisations such as the Heart Foundation and Aboriginal Community Controlled Health Organisations peak bodies, guided us throughout the study. Importantly, the National Reference Group also included representatives of ten Aboriginal Health Services from across Australia. Reference Group members attended three face-to-face meetings and also provided online feedback as the study proceeded.

One of the key outcomes of this National Reference Group was the identification of nationally and internationally recognised values and assumptions that underpin the work of the Wellbeing Study. These included the ***Declaration of Alma-Ata,*** [17] the ***United Nations Declaration on the Rights of Indigenous Peoples*** [18] and the ***Ottawa Charter for Health Promotion*** [19]. In addition, the vision of the ***National Aboriginal Community Controlled Health Organisation*** [20], which seeks to “deliver holistic and culturally appropriate health and health related services to the Aboriginal community” (p.6) was a key guiding document for the study.

The overall approach we took was based on the principles of Participatory Action Research [21] in that we established equal partnerships whereby the researchers, Reference Group members and the primary healthcare service sites collaboratively developed the study objectives and methods. Local knowledge was valued and respected, and significant time was set aside to reflect on and contribute to study methods.

### Stage one – Learning from the past

Acknowledging the importance of work that had already been completed, we first reviewed evidence from completed KVC studies [12, 13] to identify the findings that could inform this study. The National Reference Group then identified publications, reports and other grey literature that might inform the ways in which primary healthcare services have or could support the wellbeing of Aboriginal and Torres Strait Islander peoples. Altogether 97 publications were collected as part of this stage including research, program and annual reports as well as journal articles. We then undertook three systematic reviews. The first two aimed to identify the elements of existing chronic care models delivered in primary healthcare settings and to determine whether these elements were acceptable and effective [22]. The third systematic review focused on facilitators and barriers to implementing chronic disease interventions for Indigenous peoples within Australian, New Zealand, Canadian and United States primary healthcare settings [22].

### Stage two – Emerging principles

In Stage Two we used a framework analysis [23] technique to interrogate findings from Stage One. Two primary questions guided the analysis process – *How do Aboriginal and Torres Strait Islander peoples understand wellbeing?* and *How can primary healthcare services support these understandings?*

After importing all of the relevant literature into a qualitative analysis software package (QSR International’s NVivo 10 software), the entire team coded to these framework questions. The content of each code was then inductively analysed to extract answers for each of the framework questions, which became a set of draft principles. National Reference Group members were asked to review these principles at a face-to-face meeting. While members did agree on 29 draft principles as a basis for further consultation, they also expressed concern about the number of principles included, suggesting that some principles did not specifically relate to wellbeing but instead spoke more generally to the delivery of comprehensive primary healthcare.

### Stage three – Shaping a wellbeing framework

In Stage Three we aimed to organise draft principles into a Framework of essential components that could provide primary healthcare services with guidance on how to support the wellbeing of Aboriginal and Torres Strait Islander peoples living with chronic disease. Seven Aboriginal Health Services nominated at least one and in some cases two Aboriginal or Torres Strait Islander healthcare providers to participate as a Research Fellow in Stage Three. Each of the nominated Research Fellows (*n* = 13) in our team had significant experience, ranging from 5 – 14 years, in providing care to Aboriginal and Torres Strait Islander peoples with chronic diseases.

Stage Three began with a week-long workshop which allowed time for all of us to review and refine the principles, develop qualitative research skills and create semi-structured interview guides which would be used to illicit feedback from primary healthcare providers and community members on the draft principles. The week began with a presentation and explanation about why each of the original 29 draft principles identified in Stage Two had been included. Considerable debate then ensued, primarily driven by the new Research Fellows in our team, about which of the draft principles identified were relevant and applicable to their primary healthcare service. Similar to the views of the National Reference Group, we identified some principles that were less relevant or practical, while others could be either combined or deleted. This left 13 draft principles within the draft Framework for review by and feedback from primary healthcare providers and community members.

We then reviewed ethical approaches to conducting research with Aboriginal and Torres Strait Islander communities [24] with members new to research able to learn from those more experienced. Time for practice within a safe, supported space was also built into the week. Finally, we all participated in the development of two semi-structured interview guides – one for primary healthcare providers and one for community members. These guides were refined after piloting within the team and then again with non-team members.

During the subsequent eight weeks, the Research Fellows in our team led individual and group semi-structured interviews with a convenience sample of colleagues and clients (either currently living with or a carer of someone living with chronic disease) within their healthcare service who expressed an interest and provided informed consent to critique and provide feedback on the draft Framework. Core research staff members of the team travelled to the various sites to provide assistance when necessary and were also available by telephone and email during the data collection phase. After initial introductions and ensuring that voluntary informed consent had been obtained, community members and healthcare providers who agreed to participate were provided with a copy of the draft Framework and, drawing on their own experiences, were invited to think about how the Framework could be improved. Community participants were also encouraged to talk about what “kept them strong” which was agreed upon as an appropriate starting point to explore ideas of wellbeing.

All but two individual and three group interviews were audio recorded and transcribed by an external transcription service. Where audio recordings were not capture, we collected extensive field notes which were scribed for use in the analysis and interpretation process. Transcriptions and field notes were then de-identified prior to analysis and interpretation. A total of 72 community members and healthcare providers participated in 40 interviews across the seven participating Aboriginal Health Services (Table 1). In accordance with the Australian Standard Geographical Classification System [25], two sites were classified as RA1 Major City, one as RA2 – Inner Regional, three as RA3 – Outer Regional and one as RA4 - Remote (Table 1).

**Table 1:** Description of participants for Stage Three

Region	Aboriginal or Torres Strait Islander Peoples	Non-Indigenous	Total
RA1 Major City			
Community	12	0	12
Healthcare Provider	7	3	10
RA2 Inner Regional			
Community	5	0	5
Healthcare Provider	4	0	4
RA3 Outer Regional			
Community	12	0	12
Healthcare Provider	11	6	17
RA4 Remote			
Community	0	0	0
Healthcare Provider	9	3	12
Total	60	12	72

At the end of the eight week data collection phase we all came together once again with the aim of thematically analysing and interpreting the data. This second week began with a workshop on qualitative analysis and interpretation techniques. Each Research Fellow, with the assistance of core research staff, then manually coded the transcripts and field notes identifying the emerging themes which suggested how the draft Framework could be improved. In order to ensure that the context of the interviews was not lost in this process, Research Fellows participated in coding and then interpreting data from their own site.

### Building consensus

Finally, National Reference Group members together with our whole team attended a two day Consensus Workshop to review and finalise the core values, elements and principles. While suggestions for improvement were identified, including the development of a one page introduction describing the nationally and internationally recognised values and principles, agreement on the structure and content was reached.

After receiving Reference Group endorsement, our core research staff often together with a Research Fellow provided feedback to participants as well as other community members and healthcare staff at each of the participating Aboriginal Health Service sites. The responses of participating sites to these feedback meetings confirmed the outcomes from the Consensus workshop. They also identified a number of ways in which the Wellbeing Framework could be of used within their facility.

## Results

**Fig. 2:**
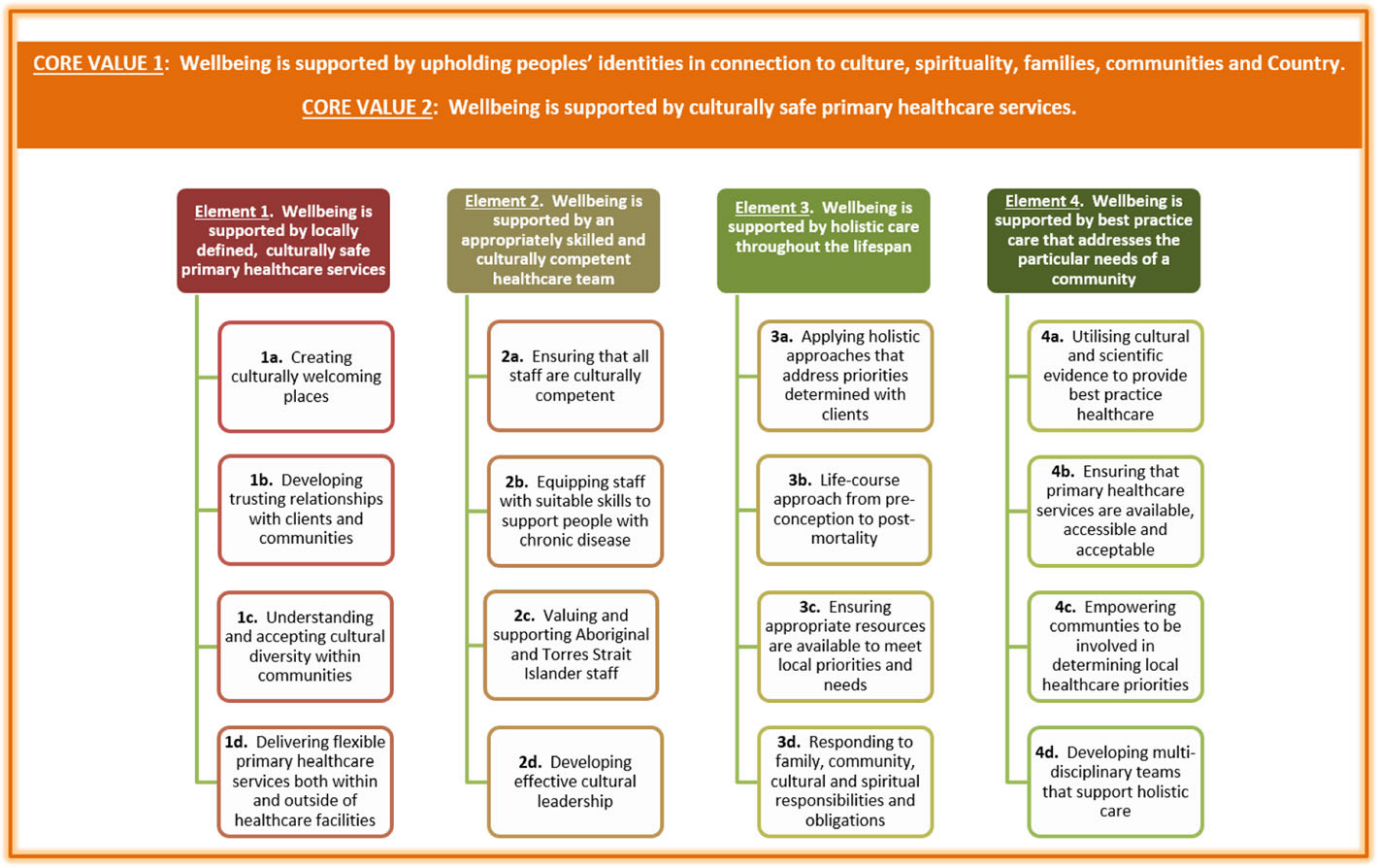
Wellbeing Framework

As a result of this complex three-stage process, two core values – ***upholding peoples’ identities in connection to culture, spirituality, families, communities and Country*** and ***culturally safe primary healthcare services*** – were identified as fundamental aspects of appropriate care for Aboriginal and Torres Strait Islander peoples. These values permeate all four key elements of the Framework – locally defined, culturally safe services; appropriately skilled and culturally competent staff; responsive, holistic care throughout the lifespan; and best practice care to address local needs. In turn, each of these four elements is supported by four principles, which further frame and provide guidance toward an approach to chronic disease care that can effectively support Aboriginal and Torres Strait Islander peoples’ wellbeing (Fig. 2).

To assist with the adaption of the framework, each of the principles are underpinned with applications suggesting how the principle could (not should) be applied in a primary healthcare setting (not shown in Fig. 1). Each of the suggested applications is referenced to specific evidence from participants in this study and/or findings from the synthesis of previously published work identified in Stage Two.

Details of these core values, elements and principles which evolved from the literature synthesis and participant interviews, and which in turn were refined through rigorous discussions at the research team and Reference Group level, are presented below. Supporting quotes provide examples of the experiences, beliefs and perceptions of community members and healthcare providers that were relayed during the interview process. In order to contextualise and signify key participant characteristics, codes (Table 2) are used at the end of each quote.

**Table 2:** Participant Coding

Ethnicity	Aboriginal and/or Torres Strait Islander = Aboriginal
	Non-Indigenous = Non-Indigenous
Role	Community participant = Community Member
	Healthcare provider participant = Healthcare Provider
State	New South Wales = NSW
	Queensland = Qld
	South Australia = SA
	Northern Territory = NT
	Western Australia = WA
	Australian Capital Territory = ACT
Location	Major City = RA1
	Inner Regional = RA2
	Outer Regional = RA3
	Remote = RA4

Finally, one of the many identified applications suggesting how a particular principle could be applied in a primary healthcare setting is presented.

### Core values

#### Wellbeing is supported by upholding peoples’ identities in connection to culture, spirituality, families, communities and country

The first core value identified by participants related to the importance of upholding peoples’ cultural connectedness and balance within their families, communities, Country, culture and spirituality. These beliefs were believed to shape people’s lives, as well as their spirituality, values, attitudes, concepts, language and relationships to the physical and material world.

[T]he thing, too, is that when you’re connected to land or country, ocean or water, you’re connected spiritually, because they’ve got spirit in it. When you’re happy inside you – that’s where wellbeing comes from. [Aboriginal, Community Member, NSW RA1].

#### Wellbeing is supported by culturally safe primary healthcare services

The second core value that emerged related to the need to ensure the cultural safety of people using service. According to participants, cultural safety included a deeper level of interaction and thoughtful practice that ensures safe services, as defined by those who *received* services.

Because I think the place itself needs to be a safe place, a place to just have something to say that. I think it needs to be something where our community feels safe within the organisation, whether it’s AMS [Aboriginal Medical Service] or mainstream. [Aboriginal, Healthcare Provider, NT RA3].

### Element 1: Wellbeing is supported by locally defined, culturally safe primary healthcare services

The first of four elements included within the Wellbeing Framework focused on the facility. The importance of the primary healthcare space, encompassing the principles of culturally welcoming places; trusting relationships; support for cultural diversity; and flexible service provision were identified as essential to supporting the wellbeing of Aboriginal and Torres Strait Islander peoples living with chronic disease.

#### Principle 1a: Creating culturally welcoming places

According to participants, primary healthcare spaces should be welcoming. This included not only the physical spaces but also the staff within the facility actively working towards ensuring that Aboriginal and Torres Strait Islander community members feel culturally safe and acceptable.

If people are going to be accessing services they need to be able to feel comfortable and welcomed into a place. And often the way to do that is to make sure that it is appropriate for them in a cultural way. So that’s around, not just the physical locality in terms of, you know, making it look like a welcoming place, by using cultural artefacts and paintings and colours. But also by having Aboriginals on staff, working in the service to actually be there to support them and provide them with the service. [Aboriginal, Healthcare Provider, Qld RA3].

This could be achieved by engaging with Aboriginal and Torres Strait Islander communities to determine what constitutes safe and welcoming healthcare spaces within their local context [26].

#### Principle 1b: Developing trusting relationships with clients and communities

Participants also identified the need for healthcare providers to develop trusting relationships with clients and communities. Communicating responsively and responsibly and ensuring that Aboriginal and Torres Strait Islander peoples feel respected, valued and cared was fundamental to the development of trusting relationships**.**

It takes time to build up the trust, so, workers that have been around for a long time contribute to that – that makes the patient more comfortable, more accessible and – I work in chronic disease out in the community, so a lot of mine is done at home in the person’s yard or house, so, you quickly build a rapport and relationship with the patients. [Aboriginal, Healthcare Provider, NSW RA3].

This could be achieved through the allocation of appropriate case loads ensuring that staff have sufficient time to build and maintain relationships with clients [27].

#### Principle 1c: Understanding and accepting cultural diversity within communities

The need to acknowledge and take account of the diversity between and within Aboriginal and Torres Strait Islander communities also featured as a key theme which, according to participants, was often overlooked. Different communities and groups within communities have distinct laws, governance arrangements, kinship structures and ways in which they view and maintain cultural identities and which, by extension, shape how health and wellbeing is framed, sustained and enabled.

What you’ve got to remember is that Aboriginal people are so diverse. It’s not – it’s not going to be one model that fits all and that’s going to be the issues. Ah, in particular as far as language barriers, cultural barriers, everything else you need to take into consideration. [Aboriginal Healthcare Provider, SA RA1].

This could be achieved by involving local community members in the development of culturally safe practices [28].

#### Principle 1d: Delivering flexible primary healthcare services both within and outside of healthcare facilities

In order to adequately meet the complex needs and competing demands experienced by some Aboriginal and Torres Strait Islander communities, participants believed the provision of services should extend beyond the geographical and time constraints which are often applied in conventional primary healthcare settings. The quote from a healthcare practitioner below encapsulated the views of other participants who cautioned against a rigid healthcare system which constrains services to one particular time or place.

[A] rigid application of the rules, so to speak, is actually damaging someone’s healthcare […] we still have people who won’t come, we still have people who won’t access the service… So now we’ve got the mobile bus – we’ve got a healthcare bus so, it’s almost like, we’ve done everything we can to get people in, now maybe we have to go out. So we’ve been doing that and I just don’t mean like immunisations and stuff, we’ll actually do healthcare clinics in the bus. [Non-Indigenous Healthcare Provider, NSW RA3].

This could be achieved by taking healthcare services out of the clinic into peoples’ homes, schools, cultural venues and parklands [29].

### Element 2: Wellbeing is supported by an appropriately skilled and culturally competent healthcare team

The second element of the framework identified the importance of the primary healthcare team who are suitably skilled and regarded by the community as culturally competent. Participants also highlighted the importance of valuing and supporting Aboriginal and Torres Strait Islander staff; and the need for effective cultural leadership.

#### Principle 2a: Ensuring that all staff are regarded by the community as culturally competent

Ensuring all primary healthcare staff are culturally competent helped to protect the rights and safety of clients. While cultural competency was often mentioned in the interviews, the following non-Indigenous healthcare provider explained the importance of support from people who could act as a cultural mentor.

So, for me, it’s about finding a mentor who can help me understand what people are going through because, you know, I have no concept of what – I don’t live those – the same lives as some of our patients and, so, I have no concept about some of, you know, a lot of what people are going through. So, I think, having those senior respected people who can guide you. [Non-Indigenous, Healthcare Provider, WA RA4].

This could be achieved by involving Elders and other members of local Aboriginal and Torres Strait Islander communities in the development and provision of cultural training [30].

#### Principle 2b: Equipping staff with suitable skills to support people with chronic disease

Enhancing the professional development of staff ensured the currency of clinical skills and encouraged retention of the primary healthcare workforce, thereby supporting continuity of care. Participants also acknowledged the need for staff to have the skills necessary to appropriately care for the complex needs of people living with chronic disease.

The qualifications and cultural competence have to go hand in hand. I guess one of the things that we sometimes have difficulty, in even as an Aboriginal and Islander organisation, yes, we want to get the cultural competence, and often the skills are lost. Or if you go with the skills you lose that [cultural competence]. We have difficulty trying to get a good balance between the two of them. One of the things that this organisation has done to improve, that is to help our staff get the skills and get the qualifications they need because it’s a lot easier to get someone who has – is culturally competent and knows the community and do all the engagement with the community because that’s what we do. [Aboriginal, Healthcare Provider, Qld RA3].

This could be achieved by developing recruitment policies that ensure that potential staff have sufficient skills, understanding and ability to contribute to the healthcare needs of communities [31].

#### Principle 2c: Valuing and supporting Aboriginal and Torres Strait islander staff

As a result of their cultural understandings and community connections, participants agreed that Aboriginal and Torres Strait Islander staff bring important unique contributions and perspectives to the primary healthcare team. As many Aboriginal and Torres Strait Islander staff who live in the community don’t “stop at 5 o’clock when the doors close” [Aboriginal, Healthcare Provider] there is often a need to extend support for these workers in particular. Participants were also quick to point out the need to see the role of Aboriginal and Torres Strait Islander staff as more than just a gateway to the community.

And normally what they [mainstream] do, as I said earlier, they would employ an Aboriginal person to be their community engagement officer. But they wouldn’t see them as being someone that they could build the skills up on to take on one of those other roles because they see those things as their responsibility and – in relation to their role. […] So non-Indigenous organisations see that community development, engagement officer role as an Indigenous position but I don’t think they recognise the importance of making sure that there’s some sort of career pathways for people so they can move on. [Aboriginal, Healthcare Provider, Qld RA3].

This could be achieved by including Aboriginal and Torres Strait Islander staff in setting primary healthcare priorities and in decision making within the primary healthcare service [32].

#### Principle 2d: Developing effective cultural leadership

Effective leadership qualities essential for guiding the primary healthcare service included the ability to understand and meet the diverse needs of communities. Ensuring people in management and governance positions have the capacity to envisage the way forward and provide a transparent system of governance was discussed at length by a number of participants.

Governance is a huge issue, and has been a huge issue, in a lot of different, community services. And it’s one thing to – to have the training around governance, but how do you ensure that it’s upheld? So, you know, that goes towards leadership as well, and making sure that you have – that you have strong leaders that can uphold, that governance, and be – not be swayed by outside forces or outside influences. […] I do feel that community, needs to sit alongside governance and leadership, because where are you going to get that leadership from? And it’s, you can say to a community, “Here’s your leader,” but if they’re not recognised by the community, then, you know, they’re not really going to be that effective. So community needs to be at the forefront, of all these decision-making processes. [Aboriginal, Healthcare Provider, SA RA1].

This could be achieved by supporting local community members to actively guide and govern local primary healthcare services [32].

### Element 3: Wellbeing is supported by holistic care throughout the lifespan

The third element of the Wellbeing Framework focuses on the breadth of care. In particular, the importance of responsive, holistic care throughout life, approaches relying on resourcing for local needs; and responding to complex responsibilities and obligations were all considered essential to support the wellbeing of Aboriginal and Torres Strait Islander peoples living with chronic disease.

#### Principle 3a: Applying holistic approaches to address priorities determined with clients

Discussions pertaining to the holistic approaches highlighted a need to address the physical, spiritual, social, emotional, psychological and cultural aspects of Aboriginal and Torres Strait Islander peoples’ health.

The way that we look at health and wellbeing it’s around, not just being about you being physically unwell, it’s about your social and emotional, mental health needs, your cultural health needs, your spiritual health needs, all of those things that support your wellbeing. So any model that’s developed has to be thinking about that in the context of okay, it can’t just be about because you’ve got a chronic disease, we need to manage your chronic disease. Because people who have – chronic disease is one part of a whole wellbeing, the whole part of a person. So that cycle of care needs to be thinking about how you actually support a person and all their needs. [Aboriginal, Healthcare Provider, NT RA3].

This could be achieved by encouraging people to become involved in their own healthcare and then responding to needs and priorities determined with clients [33].

#### Principle 3b: Life-course approach from pre-conception to post-mortality

The development of risk factors for chronic diseases was believed to be influenced by parents’ health prior to conception as well as during pregnancy. Likewise, the ongoing responsibilities for Aboriginal and Torres Strait Islander peoples who have passed, together with higher rates of morbidity and mortality, may result in an increased burden of unresolved grief, loss and trauma. A cycle of care which acknowledges that Aboriginal and Torres Strait Islander peoples’ needs differ according to where they are within their life-course was discussed.

Because when you get diagnosed of that particular disease or a chronic illness, your journey is not going to stop and start at that particular time, that you said you feel all right at that particular point. Because that’s why you are given medication and the education, awareness, that you have to go through on that journey is all part and parcel of that rehab or therapy or whatever thing that you go through. That’s a continuous journey. It shouldn’t stop, but you should also have a starting point for it to happen, too, so that they know from the beginning that when they, at first point of contact, that that journey is going to be a long journey, it’s not going to be a short journey any time soon, or it’s not going to end any time soon. [Aboriginal, Community Member, Qld RA3].

This could be achieved by developing and implementing age specific disease management and prevention, and health promotion programs [34].

#### Principle 3c: Ensuring appropriate resources are available to meet local priorities and needs

Ensuring the availability of appropriate resources to meet the often complex needs of Aboriginal and Torres Strait Islander clients was considered important. Participants spoke about developing and tailoring resources that met the specific needs of their local communities.

[I]n terms of looking at what resources, or what you might be looking to develop or get brought into your areas, consulting with the community and seeing what their needs are, and sort of that to some extent should be driven by them. But, trying to draw obviously on local resources and locally developed themes, rather than getting outsiders coming in and trying to deliver services when they may not understand the community, as well as people that live there. [Aboriginal, Healthcare Provider, NT RA3].

This could be achieved by developing a directory of local services appropriate for the needs of Aboriginal and Torres Strait Islander peoples.

#### Principle 3d: Responding to family, community, cultural and spiritual responsibilities and obligations

Participants acknowledged that wellbeing for Aboriginal and Torres Strait Islander peoples is closely connected to cultural practices, as well as to the maintenance and application of traditional knowledge. Primary healthcare providers who understood and were willing to respond appropriately to the range of cultural responsibilities, including family and kinship obligations were required.

[T]here’s some [communities] out there that still have cultural practices and an Aboriginal person might go and see a doctor and say, my uncle’s passed away, I just saw him last night and I’ve cut myself. The doctor would automatically think this guy’s psychotic or medicate him or put him in but they’re not looking at cultural triggers, they’re not looking at the environment that he’s living in, is that tribe still practising those practises. So it might be sorry cuts, it might – we believe in seeing our spirits and them guiding us. [Aboriginal, Healthcare Provider, NSW RA1].

This could be achieved by actively seeking Aboriginal and Torres Strait Islander staff members’ advice in order to give context to the circumstances of clients’ families or their communities [35].

### Element 4: Wellbeing is supported by best practice care that addresses the particular needs of a community

The final element of the Wellbeing Framework considered the type of care provided. Best practice care was considered to be one that values cultural as well as scientific evidence; ensures that care available, accessible and acceptable; empowers communities to be involved in determining local priorities; and develops multidisciplinary teams for chronic disease care.

#### Principle 4a: Utilising cultural and scientific evidence to provide best practice healthcare

While best practice care is usually based on the use of evidence from well-designed and conducted research based on a western paradigms, participants acknowledged that many community members also hold a the strong belief in Aboriginal and Torres Strait Islander healing practices. Considering the use of Aboriginal bush medicines, traditional healers (e.g. Ngangkari) and formal or informal ceremonies that reaffirmed people culturally or spiritually was also believed to be an important aspect of care.

Especially if it was someone that’s got a chronic condition where they feel like all they’re taking is medicine after like, a box of medicine a day like sometimes just having something where you go okay, let’s do a support, why don’t we do a day where we just get out and we’ll go down to the land, go down to country and you can put your feet on your country again and you know, centre yourself and hopefully that will help them or going out and doing bush medicine or doing ceremonies like whatever’s going to make them feel I guess connected spiritually and culturally again. Sometimes that can help as well. [Aboriginal, Healthcare Provider, SA RA1].

This could be achieved by the inclusion of traditional healers as part of the chronic disease team [28].

#### Principle 4b: Ensuring that primary healthcare services are available, accessible and acceptable

Participants suggested that by exploring with communities the factors that impede peoples’ engagement with healthcare services, primary healthcare services can implement strategies to increase the availability, accessibility and acceptability of care in order to adequately meet local needs.

And it’s like – like my dad’s probably, fortunate, he’s got 10 children, and each of us can take turns to try and get him in a taxi and bring him here. But for people that don‘t have that sort of support, and especially if they’re by themselves, and they’ve got an appointment here, and they live in [name of town removed], they are not going to, you know, on their own, worry about getting a bus or sorting out those networks. [Aboriginal, Community Member, Qld RA3].

This could be achieved through the provision of transport to primary healthcare facilities including vehicles that can accommodate people with limited mobility [30].

#### Principle 4c: Empowering communities to be involved in determining local healthcare priorities

Encouraging open and continuous dialogue between communities and primary healthcare providers, and ensuring that communities are able to make informed decisions about the type of care that is needed, was also a theme which emerged during data collection. Fostering a sense of empowerment was one strategy which participants believed would support the wellbeing of entire communities.

[I]t’s a matter of listening, you know that thing of listening to your community, and really listening to your community, not – it – not, you know – it’s, like, the wider community at the moment, obesity, and things like that is what the – the – the Australian Government and things like that are – are pushing, but our community, it’s, you know, mental health. Drug and alcohol is a big thing, you know, these are things that need to be addressed, yet because of grants that are only specific – you know, you’ll get a grant if you trap – tackling obesity, you know. Healthcare, right across the board, needs to be – you know, you need to let people use the – what’s avail – what their community needs, to make them move forward, is how you’re going to get stronger people. [Aboriginal, Community Member, ACT RA1].

This could be achieved through the facilitation of regular dialogue and continuous consultation between primary healthcare providers and communities [36]

#### Principle 4d: Developing multi-disciplinary teams that support holistic care

Finally, the participants in this study acknowledged the complex interplays between physical, social, emotional, and spiritual aspects of health, expressing that multi-disciplinary teams were needed to adequately address the interplay between health and wellbeing.

You’ve got to have the best staff, adequate resources and you know, a comprehensive healthcare team that, you know, that, in terms of allied health support, dieticians, diabetes educators, exercise physiologists, you know, the dentists, there’s all these different areas that, you know, that all of those different people that absolutely have a massive vital role. [Non-Indigenous, Healthcare Provider, NSW RA3].

This could be achieved by co-locating healthcare providers including traditional healers, complementary health practitioners, pharmacists, psychologists, social workers, drug and alcohol workers, allied health staff, and non-clinical support workers.

## Discussion

This study has led to the development of a framework to assist primary healthcare services to improve the wellbeing, as well as the health outcomes, for Aboriginal and Torres Strait Islander peoples living with chronic disease. The final Wellbeing Framework, consisting of two core values and four elements supported by a number of principles and applications, has several key strengths. First and foremost, the Wellbeing Framework was developed by and for Aboriginal and Torres Strait Islander peoples. A team of researchers including thirteen Research Fellows, who were also experienced healthcare professionals working in Aboriginal Health Services across Australia, came together to undertake this important work. The National Reference Group that guided the entire study included Community Elders, as well as Aboriginal and Torres Strait Islander and non-Indigenous policy makers, healthcare providers and administrators. Over 70 community members and healthcare practitioners who provide care to Aboriginal and Torres Strait Islander peoples contributed to the research findings by participating in semi-structured interviews during Stage Three of the study.

### Developed by and for aboriginal and Torres Strait islander peoples

The connections that the Research Fellows have with their local communities as well as to other healthcare providers were crucial for ensuring the acceptability and utility of this Wellbeing Framework. As has previously been identified [37], researchers who are embedded within participants’ communities are better able to understand the values and life experiences of people within particular groups. This has the added benefit of facilitating a greater acceptance of researchers and strengthening rapport with participants [38]. It is important to note that the Aboriginal and Torres Strait Islander team members did not just collect the data but were involved in the study from developing the research questions, protocols and data collection tools, to co-facilitating interviews, and then analysing and interpreting the data. This ensured that a deeper understanding of the context within which the participants’ stories were being told informed the final Wellbeing Framework [39].

Given the complex family and community relationships and the connections with Country that exist within many communities, it was important to apply a methodology that valued and privileged the knowledge of Aboriginal and Torres Strait Islander peoples in order to fully identify what is needed to understand and support the wellbeing of Aboriginal and Torres Strait Islander peoples [40, 41]. In addition to contextual and cultural knowledge, the Research Fellows also had the necessary experience in providing care and collegial relationships with other healthcare providers who were invited to participate in Stage Three of this study.

### Flexible approaches to wellbeing

Rather than defining what wellbeing is, or rigidly determining how care should be provided, the outcome of this collaborative effort is a framework that encourages locally relevant, flexible approaches to healthcare. This flexibility is particularly important given that the concept of wellbeing is difficult to define and even harder to measure [42]. Terms such as health, quality of life and ‘wellness’ [43] have all been used interchangeably. One reason for this is the number of different disciplines and perspectives, including psychology, social epidemiology, public health and medicine, which are grappling with the wellbeing concept. Another reason is that wellbeing is subjective, dependent to some extent on a person’s lived experiences, the people they associate with and the context in which they reside [44].

Given the vast number of Aboriginal and Torres Strait Islander populations across Australia the differences between how people experience wellbeing are important to consider. For example, wellbeing for Nywaigi peoples who are traditional owners in northeast Queensland is closely associated with a relationship to their ancestral lands and their ability to participate in resource management activities [45]. For the Yaegl tribe of northern New South Wales, spirituality and wellbeing were closely connected [46], while in Central Australia a study involving younger Aboriginal men found that wellbeing was closely tied to “the Law [Tjukurpa], family [Walytja], the land [Ngurra], and the sense and obligations to care for and remain connected to the social, physical and emotional world around them [Kanyini]” ([47], p. 5). Therefore, rather than defining wellbeing, this Framework seeks to encourage primary healthcare services to engage with communities and patients in order to develop a locally relevant model based on this Framework.

### Practical solutions

A key strength of this Wellbeing Framework is that it suggests practical ways to apply each of the principles. These applications are not only based on synthesis of previously published work, but also upon the contributions of community members and healthcare providers, who were actively engaged in evaluating whether they believed the applications identified within the existing literature would be effective and acceptable for use within Aboriginal Health Services. Participants also identified examples from their own experience of how these principles might be applied. Identifying applications which could support the implementation of principles will assist primary healthcare services to start to operationalise the Wellbeing Framework. Importantly,many of the applications require little or no funding, which is often a barrier to implementing new interventions [48].

In addition to assisting primary healthcare services to support the wellbeing of Aboriginal and Torres Strait Islander peoples, there are a number of other uses for the Framework. For example, some of the participating Aboriginal Health Service sites are already using it as a foundation for evaluation frameworks to monitor and learn from current programs and services. Given that the content of the Wellbeing Framework is extensively referenced, other sites have considered how it could be used to support funding applications and advocate for policy change.

### Mutually beneficial capacity strengthening

Finally, there were mutually beneficial outcomes for all members of the research team and ample examples of both-way learning from the research process [49]. The original core research staff learnt about the contextual complexities involved with providing care, while the Research Fellows had opportunities to strengthen their capacity to undertake qualitative research. For the latter, these research skills were also of direct benefit to their Aboriginal Health Service in so far as the Research Fellows now have the skills to be able to not only contribute to other research projects but also to assist with evaluation and continuous quality improvement programs. These ‘learn-by-doing’ components of the study went beyond merely data collection to developing research tools, obtaining informed consent as well as analysing and interpreting the data that had been collected.

### Limitations

While the Wellbeing Framework suggests a number of practical applications for each principle, additional work is needed to identify and, where necessary, develop resources to ensure that primary healthcare services can make full use of these applications. In the case of ***Principle 1a: Creating culturally welcome places*** an appropriate list of interpreting services for all available Aboriginal and Torres Strait Islander services could be developed and made available through a website. Health promotion materials which have been specifically designed for Aboriginal and Torres Strait Islander populations could also be identified and contact details for the developer made available. In addition, while designed to be flexible and adaptable for local community use, it is not yet known if the Wellbeing Framework is universally applicable.

## Conclusion

This study has developed a Wellbeing Framework which will assist primary healthcare services to improve the quality of care, as well as the health outcomes, for Aboriginal and Torres Strait Islander peoples living with chronic disease. Our team of researchers including thirteen Research Fellows, who were also experienced healthcare professionals working in Aboriginal Health Services across Australia, came together to undertake this important work. Similar to other studies which have used a Participatory Action Research approach, this Wellbeing Study actively engaged people with local knowledge and experience.

One of the key strengths of this Wellbeing Framework is that rather than defining what wellbeing is, or rigidly determining how care should be provided, the outcome of this collaborative effort is a Framework that allows for more locally relevant, flexible approaches by identifying key principles necessary for supporting healthcare services to respond to a community’s and individual members’ understandings of wellbeing. Importantly, the Wellbeing Framework that we developed also includes practical examples of how the principles could be applied based on the work of other researchers as well as experiences of community members and healthcare providers that participated in this study.

## Abbreviations

AMS: Aboriginal Medical Service
KVC: Kanyini Vascular Collaboration
NHMRC: National Health and Medical Research Council
